# Generating libraries of iTol2-end insertions at BAC ends using *loxP *and *lox511 *Tn10 transposons

**DOI:** 10.1186/1471-2164-12-351

**Published:** 2011-07-07

**Authors:** Leighcraft A Shakes, Gembu Abe, Mugtaba A Eltayeb, Hope M Wolf, Koichi Kawakami, Pradeep K Chatterjee

**Affiliations:** 1 Julius L. Chambers Biomedical/Biotechnology Research Institute & Department of Chemistry, North Carolina Central University, 1801 Fayetteville Street, Durham, NC 27707, USA; 2 Division of Molecular and Developmental Biology, National Institute of Genetics, Mishima, Shizuoka 411-8540, Japan; 3 Department of Chemistry, University of North Carolina at Chapel-Hill, Chapel-Hill, NC 27599. USA

## Abstract

**Background:**

Bacterial Artificial Chromosomes (BACs) have been widely used as transgenes in vertebrate model systems such as mice and zebrafish, for a variety of studies. BAC transgenesis has been a powerful tool to study the function of the genome, and gene regulation by distal *cis-*regulatory elements. Recently, BAC transgenesis in both mice and zebrafish was further facilitated by development of the transposon-mediated method using the Tol2 element. Tol2 ends, in the inverted orientation and flanking a 1 kb spacer DNA (iTol2), were introduced into the BAC DNA within the bacterial host using recombination of homologous sequences. Here we describe experiments designed to determine if a simpler and more flexible system could modify BACs so that they would be suitable for transgenesis into zebrafish or mouse embryos using the Tol2 transposase.

**Results:**

A new technique was developed to introduce recognition sequences for the Tol2 transposase into BACs in *E. coli *using the Tn10 transposon vector system. We constructed pTnloxP-iTol2kan and pTnlox511-iTol2kan to introduce the loxP or lox511 site and iTol2 cassette, containing the Tol2 cis-sequences in the inverted orientation, into BACs that have loxP and lox511 sites flanking genomic DNA inserts by Tn10-mediated transposition. The procedure enables rapid generation of a large collection of BACs ready for transgenesis with the iTol2 cassette at the new end of a progressively truncated genomic insert via lox-Cre recombination. The iTol2 ends are efficiently recognized by the Tol2 transposase, and the BACs readily integrate into zebrafish chromosomes.

**Conclusion:**

The new technology described here can rapidly introduce iTol2 ends at a BAC end of choice, and simultaneously generate a large collection of BACs with progressive deletions of the genomic DNA from that end in a single experiment. This procedure should be applicable to a wider variety of BACs containing lox sites flanking the genomic DNA insert, including those with sequence repeats. The libraries of iTol2 inserted BACs with truncations from an end should facilitate studies on the impact of distal *cis*-regulatory sequences on gene function, as well as standard BAC transgenesis with precisely trimmed genes in zebrafish or mouse embryos using Tol2 transposition.

## Background

The Tol2 transposon system has been used extensively to deliver exogenous DNA into the germline of zebrafish [1-3], and more recently for successfully integrating BAC DNA into the germline of both zebrafish and mice [4]. In this method the iTol2 cassette, comprising of the minimal cis-sequences of Tol2 in an inverted orientation separated by a ~1 kb spacer, was introduced into a BAC using recombination of homologous sequences within the genomic insert DNA in the bacterial host [5].

A different strategy to modify genomic DNA cloned in BACs uses Cre-lox recombination. This method uses the bacterial Tn10 transposon to introduce exogenous DNA, including lox sites, at random locations in the BAC [6,7]. The inserted lox sites can in turn be recombined with those endogenous to the BAC vector with Cre protein to progressively delete insert DNA from either end efficiently and selectively [8]. The procedure has been used previously to deliver reporter genes and other exogenous DNA cassettes such as sequencing primer sites, mammalian cell-selectable antibiotic resistance genes and EGFP enhancer-traps precisely at the newly created ends of the DNA insert in BACs [7-9]. It is significant that the recombinases involved in this approach, namely Tn10-transposase and Cre protein, do not act upon sequence repeats and/or other recombinogenic sites in the genomic DNA insert to rearrange it. This particular characteristic makes the approach applicable to a wider variety of BACs in the public domain, including those with repeated sequences (see reference [10], for example). Here we describe methodology to efficiently place iTol2 cassettes precisely at the ends of the genomic DNA insert using either a *loxP *or a *lox511 *recombination system. Cre-recombination of the endogenous loxP or lox511 sites, a constituent of all BACs in the public domain, with the ones inserted by these iTol2-Tn10 transposons simultaneously truncates the DNA from the respective end and delivers the iTol2 cassette. Large numbers of BACs progressively truncated from either end, with iTol2 placed at the newly created end, can thus be obtained in a single experiment.

## Methods

### Construction of Tn10 mini-transposons with iTol2kan cassettes in front of either *loxP *or *lox511*

The iTol2-kan DNA cassette was inserted in front of the *loxP *or *lox511 *site in the previously described Tn10 transposon plasmids pTnMarkerless2 [11], and pTnlox511(B)markerless1 [8]. The oligonucleotides d (GGCGCGCCTGCTCGAGCCGGGCCCAAGTGATCTC) and d (GGCGCGCCTCTAGATCAGATCTAATACTC) were used to amplify the iTol2-kan DNA cassette. The 1371 bp PCR product was flanked by Asc I sites, and contained the gene for kanamycin resistance which was in turn flanked by inverted 200 bp Tol2R and inverted 150 bp Tol2L [4]. This PCR product was inserted into AT-cloning vector pCR2.1. The DNA isolated from the clones was digested with Asc I enzyme, and the purified DNA fragment inserted at the Asc I sites of plasmids pTnMarkerless2, and pTnlox511(B)markerless1. Both orientations of the iTol2-kan insert in each of the plasmids pTnMarkerless2 and pTnlox511(B)markerless1 were isolated. The mixture of plasmids with both orientations of iTol2kan will be designated as pTn*loxP*-iTol2kan and pTn*lox511*-iTol2kan.

### Functionalizing BACs with EGFP gene cassettes

BACs were functionalized with the EGFP gene in one of two ways: using EGFP enhancer-traps in BAC clone CH211-43O16 containing zebrafish APPb gene [9], or by galK-mediated recombination in bacteria for BAC clone CH211-163F17 carrying the zebrafish fgf24 gene. The fgf24 BAC was isolated in the host SW 102 after being functionalized with the EGFP gene using the latter procedure [4,5]. Because this host contains a temperature sensitive λ-prophage, the fgf24:EGFP BAC DNA was isolated and re-introduced by electroporation into the DH10B host.

### Generating BAC deletion libraries with pTn*loxP*-iTol2kan and pTn*lox511*-iTol2kan

Deletion libraries of EGFP-functionalized BACs were made with the iTol2kan-Tn10 transposons as previously described for other BACs using different Tn-10 transposons [7-12]. The transposon plasmid DNAs from pTn*loxP*-iTol2kan and pTn*lox511*-iTol2kan were each separately introduced into the BAC clones made competent using the calcium chloride procedure. Transformed colonies were selected on LB agar plates containing both chloramphenicol and kanamycin. Approximately a thousand transformed colonies were pooled [13], and low density cultures containing chloramphenicol, kanamycin and ampicillin were set up. The cultures were induced with IPTG at early log phase of growth, and incubated further for three hours. Cells were spun down, re-suspended in one-tenth volume of fresh LB in the absence of antibiotics, and infected with phage P1. Two hours into the infection, cells were treated with chloroform and lysed, the supernatant isolated and used to infect fresh NS3516 cells. These were then plated on LB agar plates containing both chloramphenicol and kanamycin. Deletion libraries generated with pTn*lox511*-iTol2kan gave better yields of un-catenated BACs if recovered from the Cre-expressing strain NS3529, instead of the Cre non-expressing strain NS3516 (PKC unpublished observations). Several thousand member BAC deletion libraries were routinely obtained using this procedure with both pTn*loxP*-iTol2kan and pTn*lox511*-iTol2kan.

### Analysis of BAC DNA from deletion libraries

DNA from BAC clones was isolated and analyzed by Field Inversion Gel Electrophoresis (FIGE) after Not I digestion as previously described [14]. The Not I sites at either end of the original BAC are deleted upon lox-Cre recombination. The Not I sites in the transposon plasmids pTn*loxP*-iTol2kan and pTn*lox511*-iTol2kan are designed such that the BAC-vector DNA band is altered in size for authentic lox-Cre recombinants [8,9]. Thus deletions with pTn*loxP*-iTol2kan result in shortening of the BAC-vector DNA band, while those with pTn*lox511*-iTol2kan become devoid of the vector band (see reference [15], and the web site http://bacpac.chori.org/ptarbac21.htm for details of pTARBAC2.1 vector used for the zebrafish BAC library). As a result lox-Cre independent deletions, and/or rearrangements, occurring within the genomic insert are easily distinguished from authentic lox-Cre recombinations originating from an end of insert DNA [8-12].

**Figure 1:**
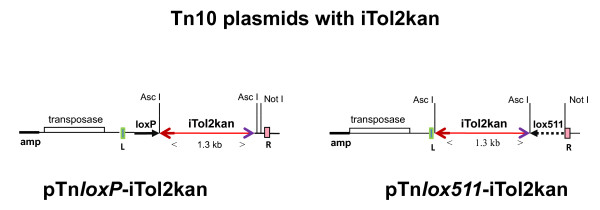
**Schematic of pTn*loxP*-iTol2kan and pTn*lox511*-iTol2kan**: The iTol2kan DNA cassette was inserted at the Asc I site in both pTnMarkerless2 and pTnlox511(B)markerless1 plasmids, in either orientation. Mixtures of plasmids containing both orientations of iTol2 kan in pTnMarkerless2, and pTnlox511(B)markerless1, were used for generating deletion libraries in BACs. Clones from the library represented both orientations of the iTol2kan in them. The vertical boxes marked R (pink) and L (green), adapted from reference [8], indicate the 70 bp inverted repeat ends of the Tn10 transposon. The genes coding for transposase and ampicillin (amp) resistance are located outside the 70 bp inverted repeat ends. The arrows, thick black and broken and not drawn to scale, represent sequences for loxP and lox511, respectively.

To identify the newly created end, BAC deletions of the desirable size were then sequenced with primers based at the ends of the Tn10 transposons retained after deletion formation, for example pink end R and green end L in deletions made with pTn*loxP*-iTol2kan and pTn*lox511*-iTol2kan, respectively (see Figure 1 and reference 8). Orientation of the iTol2kan cassette in BAC clones was determined by direct sequencing of the BAC DNA with primers located at the ends of the kanamycin gene and going outwards. These are: Tkan1: d (CTGCGTGCAATCCATCTTGTTC) and Tkan2: d (CCTTCTTGACGAGTTCTTCTG)

### Injection of BAC DNA into zebrafish embryos

DNA from chosen BAC clones were purified using the Qiagen tip procedure described earlier [7,14], and injected into zebrafish eggs as described earlier [4,9]. BACs functionalized with iTol2kan were typically injected with a mixture of 30-50 ng/uL BAC DNA plus 25-50 ng/uL transposase mRNA into one-cell stage zebrafish embryos using previously described procedures [4].

### Procedures & Primers for the "excision assay" in embryos transiently expressing EGFP

Excision assay was performed following a previously described procedure [16]. Primers used for this analysis are: kan629-outFwd: d (TCTGGATTCATCGACTGTGG) and kan186-outRev: d (GTCCTGCAGTTCATTCAGGG)

### Analysis of transgenic zebrafish

Microscopy: Developing embryos were analyzed for EGFP expression between 24 and 72 hours post fertilization, using a Nikon Diaphot Fluorescence microscope with a Nikon high pressure mercury lamp as the excitation source. Embryos were photographed with a RT Spot from Diagnostic Instruments, Michigan, USA.

## Results

We have developed novel methodology to introduce the repeat ends of Tol2 in an inverted orientation (iTol2) into BACs with high efficiency. The iTol2 DNA cassettes are placed precisely at the ends of the genomic DNA insert in BACs, while simultaneously trimming that insert DNA end by Cre-lox recombination. The end of lesser interest of the insert DNA is therefore chosen to introduce iTol2. In addition to its ease of manipulation, a key advantage of the procedure is the generation of a large collection of BACs with iTol2 introduced at the chosen end of the genomic DNA insert.

### Construction of transposon plasmids pTn*loxP*-iTol2kan and pTn*lox511*-iTol2kan

The DNA cassette, containing the 200 bp and 150 bp repeat ends of the vertebrate transposon Tol2 placed in the inverted orientation and flanking the kanamycin resistance gene (iTol2kan), was amplified by PCR. The sequence recognized by the restriction enzyme Asc I was built into the 5' end of each primer, as indicated in Methods. After sub-cloning in pCR2.1 vector, the amplified DNA was excised and ligated at the unique Asc I site of plasmids pTnMarkerless2 and pTnlox511(B)markerless1 that were described previously [11,8]. The two iTol2 transposon plasmids, each containing both orientations of the iTol2kan cassette at the Asc I site, are shown in Figure 1. Deletion libraries of BACs were generated with a mixture containing both these orientations of iTol2kan for either the loxP or lox511 Tn10 plasmids. Note that the iTol2kan cassette is placed in front of the loxP or lox511 sites in the two transposons. Thus iTol2kan would be retained in the BAC DNA after lox-Cre recombination between the transposed and corresponding endogenous lox-sites at either end of BAC genomic inserts. We have previously demonstrated that cross-recombination between loxP and lox511 sites using phage-P1 generated Cre protein during a normal phage infection is negligible [8].

### Introducing iTol2kan at the lox511 end of insert DNA in BACs

**Figure 2:**
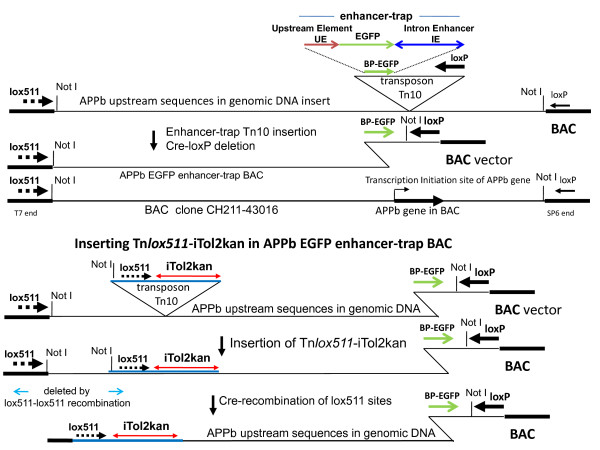
**Schematic of deletion formation in APPb:EGFP-enhancer-trap BAC by pTn*lox511*-iTol2kan**: Starting BAC APPb EGFP enhancer-trap was generated by inserting the enhancer-trap transposon (inverted triangle) is shown, along with the location of the APPb gene within the BAC clone (adapted from reference [9]). The insertion of the Tn*lox511*-iTol2kan transposon, and the resulting lox511-lox511 deletion mediated by Cre protein are shown in the lower half.

**Figure 3:**
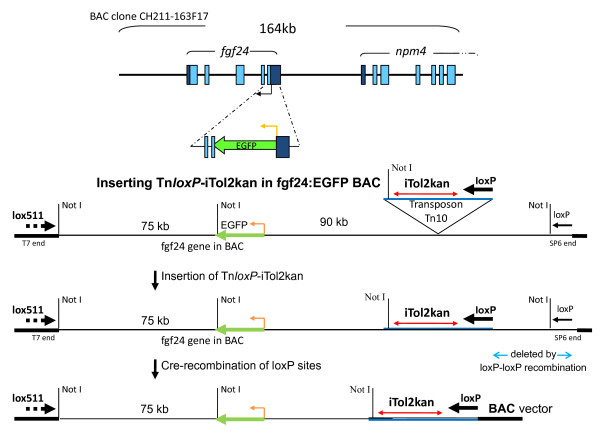
**Schematic of deletion formation in fgf24:EGFP BAC by pTn*loxP*-iTol2kan**: Layout of the fgf24 gene functionalized with the EGFP gene is shown. Below it is a schematic of the Tn*loxP*-iTol2kan transposon insertion into the EGFP functionalized BAC DNA to place iTol2kan at the loxP end of BAC insert. The loxP-loxP deletion mediated by Cre protein is indicated.

Progressive deletions of genomic DNA in BACs from either the loxP or lox511 end have been described [7,8]. An important feature of the technology is the simplicity of determining exactly where in the BAC DNA the loxP- or lox511-transposon inserted to create the truncation [7,8]. Another significant feature is the ability to precisely introduce reporter genes and other DNA cassettes at the newly created end in the large BAC clone. The sequence in front of the loxP or lox511 arrowhead as shown in Figures 2 and 3 is retained after the recombination event that creates the deletion (see also reference [9]). The arrow orientation refers to the directionality of the loxP or lox511 sequences. This particular feature is used here to place the repeat ends of the vertebrate transposon Tol2 in the inverted orientation, and flanking a kanamycin resistance gene, at either the loxP or lox511 end of the BAC DNA.

Two types of BACs from the zebrafish library were used to demonstrate the efficacy of this method: BACs containing the Amyloid Precursor Protein gene of zebrafish (APPb) functionalized with EGFP enhancer-traps as described in an earlier study [9], and a BAC containing the fgf24 gene functionalized with the EGFP gene by galK-mediated homologous recombination [4,5]. These are shown schematically in Figures 2 and 3, respectively.

**Figure 4:**
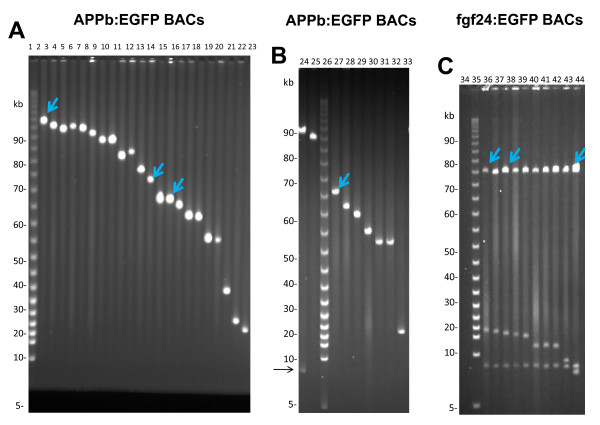
**FIGE analysis of clones from libraries generated with insertion of either pTn*lox511*-iTol2kan (panels A and B) or pTn*loxP*-iTol2kan (panel C)**. Deletion clone DNAs shown in FIGE **Panels A and B **were obtained from libraries made with different APPb BACs that contained the EGFP enhancer-trap located either 2.5 kb upstream (Panel A), or 4 kb upstream (Panel B) of the transcription start site of the APPb gene. **Panel C **shows clone DNA from the library generated with fgf24:EGFP using Tn*loxP*-iTol2kan transposon. Only clones containing an intact 75 kb fragment are shown here. All clone DNAs were digested with Not I enzyme prior to FIGE analysis. The blue arrows indicate the BAC clones tested in zebrafish for expression and/or "excision assay" analyses. All clones are numbered according to the lanes in which their DNA appears, and are referred to as such throughout the text. Lanes 1, 26 and 34 are marker lanes containing the 5 kb ladder, and BAC 24 (in lane 24) is the starting APPb BAC used to generate the deletions in panel B. The arrow at the side and bottom of Panel B indicates the position of the BAC vector DNA band, which in this case is ~7 kb. Size of DNA bands in kb is indicated on the side of each panel.

Thus a 104 kb APPb-BAC clone with a 97 kb genomic insert DNA, containing the basal promoter EGFP gene regulated by ~94 kb upstream DNA of the APPb gene, was chosen for insertion of iTol2kan at the lox511 end (Figure 2). When injected into zebrafish embryos, DNA from this and similar clones expressed GFP fluorescence exclusively in neurons [9]. Plasmid DNA from pTn*lox511*-iTol2kan was introduced into this BAC clone using calcium chloride transformation as previously described [12]. A deletion library comprising of at least 3000 clones was isolated from the iTol2kan transposon plasmid transformed BAC culture using procedures described previously [12]. DNA was isolated from clones of this library and analyzed by FIGE. Arrays of BAC deletions, each containing the iTol2kan at the lox511 end and the EGFP gene at the loxP end of genomic insert DNA, are shown in Figure 4A. Note that Cre recombination of lox511 sites, one endogenous to BAC vector and the other transposed by Tn*lox511*-iTol2kan, deletes the Not I site existing at the lox511 end of the starting BAC DNA. Consequently a separate Not I-Not I vector DNA band is not present in deletion clone DNA in the FIGE gels of panels A and B of Figure 4. Clone DNAs shown in FIGE Panels A and B were obtained from libraries made with different APPb BACs that contained the EGFP enhancer-trap located either 2.5 kb upstream (Panel A), or 4 kb upstream (Panel B) of the transcription start site of the APPb gene (see ref. [9] for details). Both orientations of the iTol2kan cassette were found in clones in the FIGE arrays shown in panels A and B of Figure 4. This was determined by direct sequencing of the BAC DNA using primers, Tkan1 or Tkan2, located at the ends of the kanamycin gene in the iTol2kan cassette and going outwards. In approximately half the clones, sequencing with either Tkan1 or Tkan2 primer helped determine the location of the newly created end in the APPb BAC made by pTn*lox511*-iTol2kan in the APPb gene region of chromosome 9 of zebrafish. BLAST analysis was used to identify these locations (shown in Additional file 1), which were consistent with the size of the BACs estimated from the markers in the FIGE arrays shown in Figure 4 Panels A and B.

### Introducing iTol2kan at the loxP end of insert DNA in BACs

The APPb BACs functionalized with EGFP enhancer-traps at the loxP end of genomic insert could not be used to introduce iTol2 cassettes again at the loxP end. Using the loxP-iTol2kan transposon on these BACs would only delete the EGFP enhancer-trap reporter cassette. Therefore a zebrafish BAC clone functionalized with the EGFP gene by galK-mediated homologous recombination was selected to introduce iTol2kan at the loxP end (shown schematically in Figure 3). The open reading frame of EGFP gene was fused to the initiator codon of the fgf24 gene in this BAC. The transposon plasmid pTn*loxP*-iTol2kan, shown schematically in Figure 1, was introduced into the fgf24:EGFP BAC using the calcium chloride transformation procedure. Note that the fgf24:EGFP BAC DNA was isolated from the host SW 102, and re-introduced by electroporation into DH10B cells to avoid potential complications arising from the temperature sensitive λ-prophage in the previous host (see Methods).

A deletion library of several thousand members was generated with fgf24:EGFP BAC using pTn*loxP*-iTol2kan, as described earlier [12]. Analysis of deletion clone DNA by FIGE after Not I digestion is shown in panel C of Figure 4. Because the EGFP cassette was introduced at fgf24 translation start site, it was important to isolate clones with genomic insert DNA longer than 75 kb (see Figure 3). Therefore only clones that fulfill this criterion are shown in the FIGE of Figure 4C.

The fgf24:EGFP BAC deletions shown in panel C were sequenced with transposon based primer, Seq 1 [7], to determine the location of the newly created end containing the iTol2kan cassette on the fgf24 BAC DNA. BLAST analysis of sequences helped determine their locations on a chromosomal map (see Additional file 1). These were consistent with the size of clone DNA in Figure 4, panel C.

### Expression analysis of iTol2-containing APPb:EGFP BAC and fgf24:EGFP BAC in zebrafish embryos

Deletion clone DNAs marked with the blue arrowheads, in Panels A, B and C of Figure 4, were further purified using the Qiagen-tip procedure [7,14], and injected into zebrafish eggs for expression and/or "excision assay" analyses along with mRNA capable of expressing Tol2 transposase [1-4].

### Excision Assay analyses to confirm authentic recognition of iTol2-ends by Tol2 -transposase

**Figure 5:**
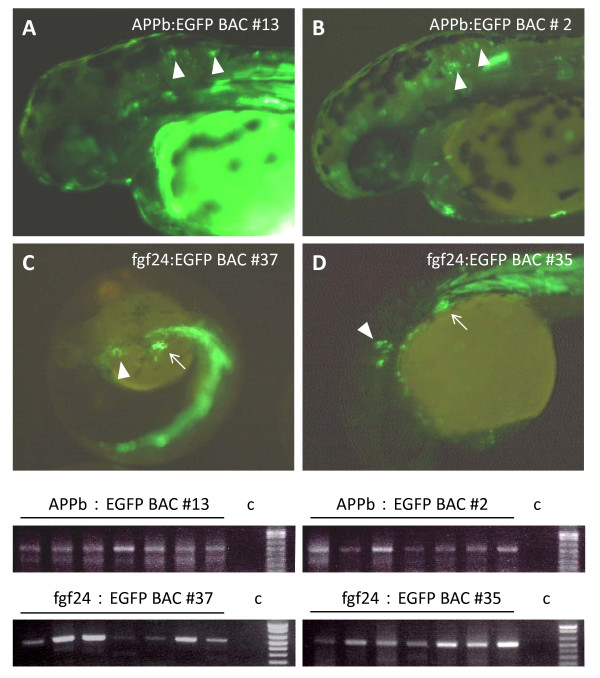
**"Excision assay" performed with genomic DNA isolated from 2-day old zebrafish embryos**: GFP expression in the embryos injected with iTol2-BACs were observed between 28 and 48 hours post fertilization (hpf) (upper panels). APPb:EGFP BAC 13 (panel 5A), APPb:EGFP BAC 2 (panel 5B), fgf24:EGFP BAC 37 (panel 5C) and fgf24:EGFP BAC 35 (panel 5D) display green fluorescence. The white arrowheads indicate the expected tissue-specific expressions of GFP in these embryos. The iTol2-BAC injected embryos were analyzed using the "excision assay" (lower panels) [16,4].

Recognition and processing of the BAC DNA, containing the iTol2kan cassette, by the Tol2 transposase enzyme upon entry into zebrafish oocytes can be evaluated using the "excision assay" [16]. Thus the embryos injected with DNA from APPb:EGFP BAC and fgf24:EGFP BAC were analyzed using the "excision assay". The results shown in the lower panels of Figure 5 clearly indicate that all of the BAC DNAs tested were processed by the Tol2 transposase. Transient expression patterns of embryos analyzed with the excision assay are shown in panels A-D of Figure 5. The results demonstrate that iTol2kan at lox511-end for BACs 2 and 13, and the loxP-end for clones 35 and 37, respectively, are recognized accurately by the Tol2 transposase. Note that BACs 2, 35 and 37 are larger than 100 kb.

### Germline expressions of iTol2 containing BACs

**Figure 6:**
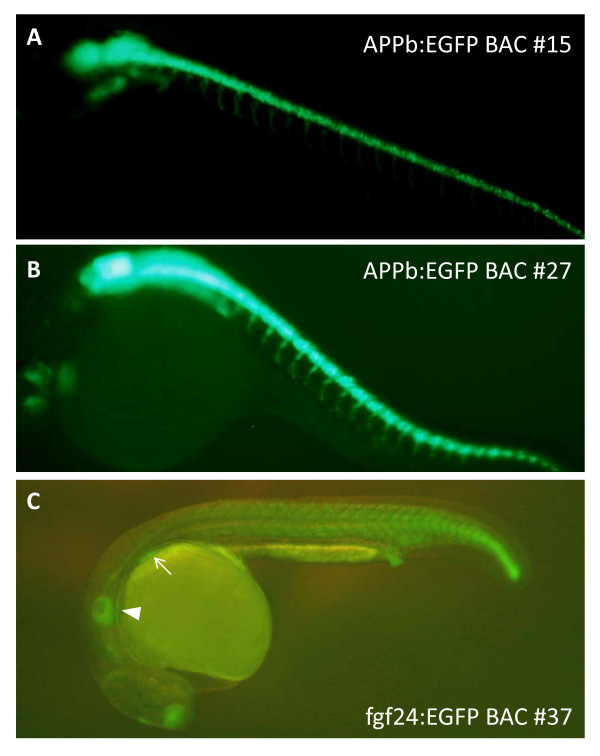
**EGFP fluorescence in transgenic F1 zebrafish**: **Panel A**: Germline expression of EGFP fluorescence in zebrafish embryos with DNA from APPb:EGFP BAC clone 15. **Panel B **shows the germline expression of EGFP fluorescence with DNA from APPb:EGFP BAC 27, and **Panel C **shows germline expression from fgf24:EGFP BAC clone 37.

Transgenic zebrafish lines were established from injections of EGFP BAC DNAs shown in lanes 15, 27 and 37 in FIGE panels A, B and C of Figure 4, respectively. The EGFP fluorescence of the F1 zebrafish lines are shown in Figure 6, panels A-C, for BAC clones 15, 27 and 37 respectively. Expression of EGFP fluorescence is restricted to neurons and highly specific to the hind brain and spinal cord in F1 transgenic zebrafish created from injections of iTol2-APPb:EGFP BACs 15 and 27, as seen in panels A and B of Figure 6. The slight difference in the expression patterns observed in the two panels might be due to the different APPb BACs used; clones 15 and 27 have DNA extending till 2.5 kb and 4 kb upstream of the APPb transcription start site, respectively.

Germline expression of iTol2 retrofitted fgf24:EGFP BAC clone 37 is shown in Figure 6C. F1 embryos from injections with fgf24:EGFP BAC 37 DNA showed GFP expression patterns almost identical to those from injections with full length fgf24:EGFP BAC DNA that had the iTol2 cassette introduced by GalK mediated recombination (5, Gembu Abe, Koichi Kawakami unpublished observations). Taken together, the results indicate that BACs larger than 100 kb are not only recognized and processed effectively by the Tol2 transposase, but are also integrated efficiently into the zebrafish chromosome when iTol2 is introduced into the BAC DNA ends using a loxP or lox511 Tn10 transposon.

## Discussion

We have developed a novel approach to rapidly introduce repeat ends of the vertebrate transposon Tol2, in an inverted orientation, at the ends of BAC DNA. The Tn10 transposons used for the purpose carry both iTol2 ends and lox sites that can be recombined by Cre protein with the corresponding lox sites endogenous to the BAC vector and flanking the genomic DNA insert. As the insertions of these iTol2-Tn10 transposons into genomic DNA in BACs appear to be largely random (see references [10,13] for discussions), large collections of BACs with iTol2 at the newly trimmed end are generated in a single experiment. Such libraries are uniquely suited for functionally mapping long-range gene regulatory sequences using transgenic animals. Thus multiple BACs from a contig spanning a genetic locus should enable such functional analyses to be extended over large regions of the genome. The approach does not require selecting sequences for mutational analyses to test their gene regulatory potential, and is therefore unbiased. It enables enhancer-trap containing BACs, or BACs retrofitted with EGFP cassettes using sequence homology based recombination, to be readily converted into deletion libraries with integrated iTol2 ready for chromosome integration. Compared to traditional approaches for enhancer-trapping used with whole genomes in animals (see references [17-25]), our approach using enhancer-traps in individual BACs has the potential to allow a more uniform coverage of the genome because the baseline efficiency of trap insertion is reset for individual BACs in the bacterial host. Although there appear to be vast regions of the genome refractory to enhancer-trapping by traditional means, to date we have not encountered BACs refractory to Tn10 transposon insertions.

The iTol2 insertions into BAC DNA are performed using Tn10 transposons carrying loxP or lox511 sites, and do not rely upon sequences existing in the genomic inserts of BACs. Consequently, the Tn10 transposons developed here are applicable to all BACs in the public domain. This feature is unlike the targeting vectors used in methods based on recombining homologous sequences, which need to be constructed anew for each BAC. The use of radioactive isotopes is also prevented, as Southern blotting is not required in retrofitting BACs using Tn10 transposons.

Varying degrees of promiscuity in recombining different mutant *lox *sites, including the lox511 mutant with wild type loxP, using both partially purified Cre-extracts *in vitro *[26-29], and Cre over-expressed in cells [30-32] have been reported. For example, cross recombination between loxP and lox511 has been reported to occur at efficiencies ranging from 5 to 100% under those experimental conditions that express Cre constitutively [26,27,29-32]. We have not observed such cross-recombination. High levels of stringency *in vivo *in recombining identical *lox *sites [8,33], or *lox *sites with at least identical spacers [34], have been achieved with Cre protein expressed from its native source, namely a phage P1 infection [8,33,34]. Depending on whether a loxP or a lox511 transposon is used, truncations from the corresponding lox- end of insert DNA in BACs can be made readily and exclusively with high stringency. Thus insertions of iTol2 with the resulting truncations of genomic DNA from either end are not only efficient, but also highly specific to that end.

A notable drawback of the transposon based approach appears to stem from the very feature that makes it so efficient: the P1 headful packaging strategy used to isolate the functionalized BAC so easily also limits the size of the BAC clone that can be analyzed to ~ 110 kb. Although the genomic DNA insert is truncated in the process to a size of ~103 kb, the remainder BAC vector, it is unlikely to be a disadvantage in most applications because a majority of vertebrate genes can be housed in their entirety within this size limit. We note that almost half of evolutionarily conserved non-coding gene-regulatory sequences in vertebrate genomes [35], and probably a similar fraction of those that are conserved in function and shape but not in sequence [36,37], are located within this span of DNA adjoining transcription start sites of genes.

It is clear that the transposon retrofitting strategy and those based on homologous recombination have strengths that appear somewhat complimentary in nature. Therefore a judicious approach might be to use a combination of the two methodologies, as demonstrated here for the fgf24:EGFP BAC and in an earlier study [38]. The procedures described here should allow one to readily generate libraries of EGFP functionalized BACs, progressively truncated from an end and carrying the iTol2 cassette, ready for integration into chromosomes of at least fish and mice [4].

## Conclusion

The repeat ends of the Tol2 transposon in the inverted orientation, and flanking a kanamycin resistance gene (iTol2), was introduced by a novel procedure to the ends of BAC DNA. We show that these iTol2 inserted BACs are efficiently recognized by the Tol2 transposase, and successfully integrate into the germline of zebrafish. Compared to previous methods using recombination of homologous sequences to introduce iTol2 ends into BACs, our lox-Cre recombination approach rapidly generates, in a single experiment, a large collection of BACs with iTol2 placed at an end that is also progressively truncated. The libraries of iTol2 inserted BACs generated should facilitate integration of trimmed single genes into the germline, and help functionally map *cis*-acting gene regulatory sequences in animals. The approach should be applicable to a wider variety of BACs, including those with sequence repeats.

## List of abbreviations

**BAC**: bacterial artificial chromosome; **FIGE**: Field inversion gel electrophoresis; **APPb**: amyloid precursor protein gene b; **EGFP**:enhanced green fluorescent protein.

## Authors' contributions

LAS screened iTol2 inserted BACs by FIGE, sequenced iTol2 BAC ends, injected BAC DNA into zebrafish embryos, and documented positive embryos using photo-microscopy. GA injected iTol2 BACs into zebrafish embryos, performed excision assays, analyzed embryos positive for expression and contributed to writing the manuscript. MAE screened libraries of iTol2 BACs, isolated clones of desirable size using FIGE and injected BAC DNA into zebrafish eggs. HMW screened and analyzed the small plasmids carrying iTol2kan and lox sites, and helped write the manuscript. PKC designed the study, helped with design and construction of the transposon plasmids, generated the iTol2 libraries of BAC deletions, purified BAC DNA for injections using Qiagen columns, and was responsible for writing the article with KK. Both KK and PKC critically evaluated the study. All authors read and approved the final manuscript.

## Supplementary Material

Additional file 1 **Location of iTol2kan insertions (new BAC ends) in APPb:EGFP and fgf24:EGFP BACs**. Newly created ends containing the iTol2kan cassette were sequenced with primers Tkan1 and Tkan2 for APPb:EGFP BAC deletions (**Panel A**), and with primer Seq1 for fgf24:EGFP BAC deletions (**Panel B**). The sequences were BLASTed to the zebrafish genome, and location of the new ends of BACs where the iTol2kan cassettes are placed indicated in **panels A and B**. Clone numbers are as in lanes shown by the blue arrowheads in Figure 4. Click here for file
