# Supplementary figures and images for: Generating libraries of iTol2-end insertions at BAC ends using loxP and lox511 Tn10 transposons
Source: BMC Genomics. 2011 Jul 7;12:351. doi: 10.1186/1471-2164-12-351 (PMC3146455; doi:10.1186/1471-2164-12-351)

## Location of iTol2kan Insertions

**A**

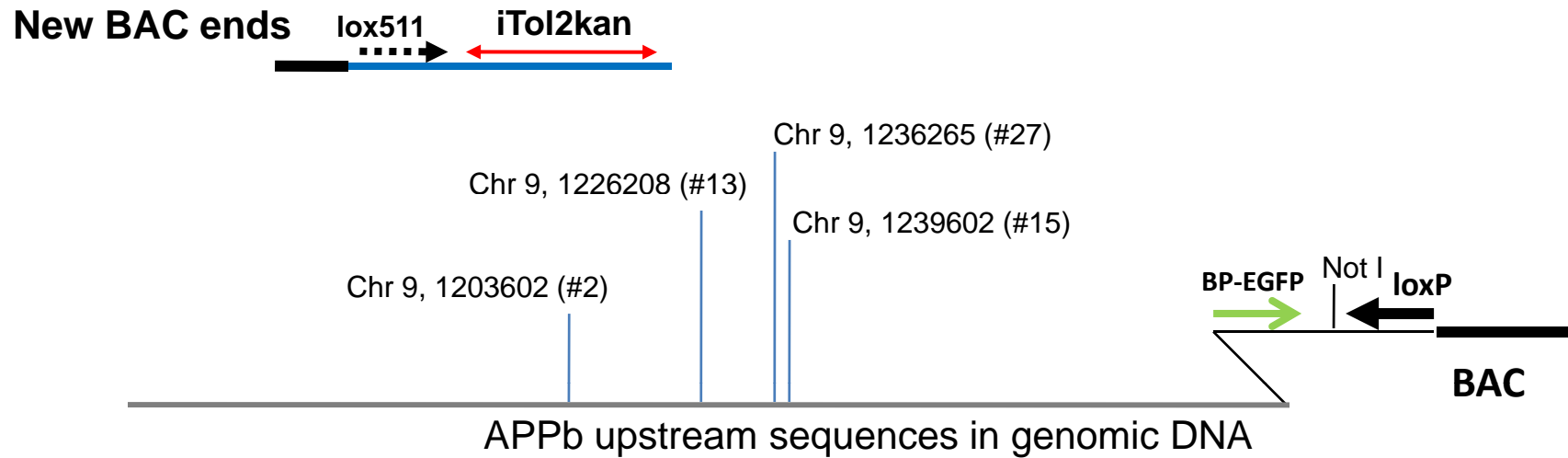

**B**

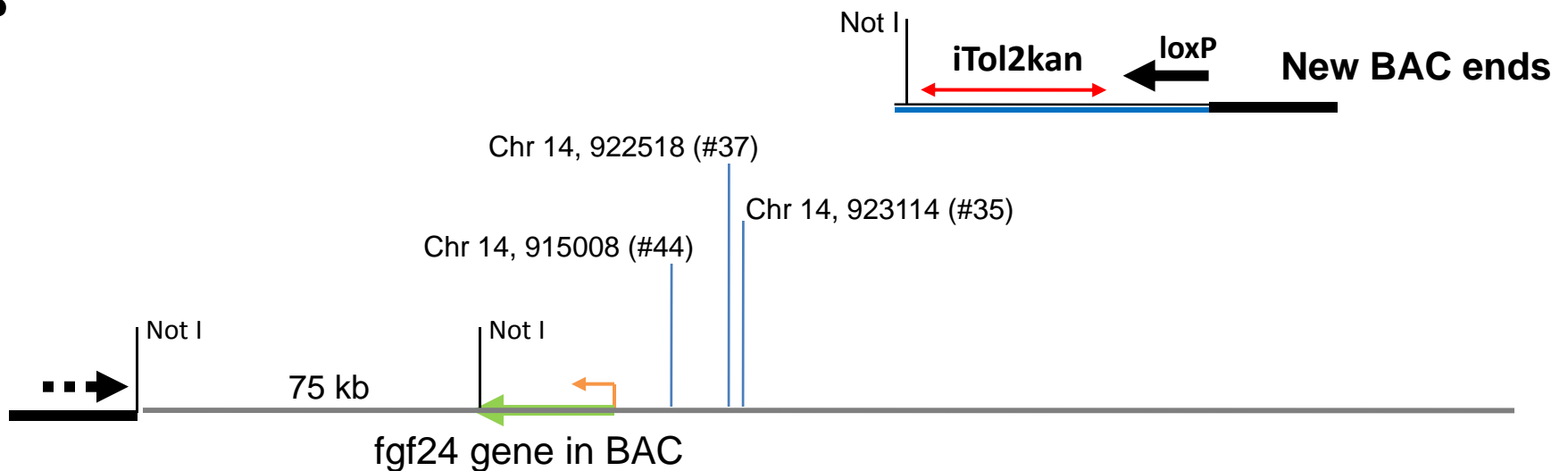

Supplement: Additional file 1 — Location of iTol2kan insertions (new BAC ends) in APPb:EGFP and fgf24:EGFP BACs. Newly created ends containing the iTol2kan cassette were sequenced with primers Tkan1 and Tkan2 for APPb:EGFP BAC deletions (Panel A), and with primer Seq1 for fgf24:EGFP BAC deletions (Panel B). The sequences were BLASTed to the zebrafish genome, and location of the new ends of BACs where the iTol2kan cassettes are placed indicated in panels A and B. Clone numbers are as in lanes shown by the blue arrowheads in Figure 4. [file 1471-2164-12-351-S1.PDF]
